# Ensuring research continuity during crises: A scoping review

**DOI:** 10.1371/journal.pone.0353082

**Published:** 2026-11-02

**Authors:** Lili Zeidan, Sarah Kamareddine, Macy Zaatar, Mohamad Sadek Zoghbi, Shahinda Hammoud, Hebatalla Shamseldin, Ola El Zein, Makram Halawani, May Awar Ammar, Elie A. Akl

**Affiliations:** 1 Clinical Research Institute, American University of Beirut, Beirut, Lebanon; 2 Faculty of Medicine, American University of Beirut, Beirut, Lebanon; 3 Office of Research, American University of Beirut, Beirut, Lebanon; 4 Leiden University, Leiden, Netherlands; 5 Harvard University, Boston, United States of America; 6 Department of Internal Medicine, American University of Beirut, Beirut, Lebanon; 7 Department of Health Research Methods, Evidence, and Impact (HEI), McMaster University, Hamilton, Ontario, Canada; Dana-Farber Cancer Institute, Harvard Medical School and THE Broad Institute of MIT and Harvard, LEBANON

## Abstract

**Background:**

During crises, both challenging circumstances and uncertainty threaten the continuity of research operations. Research projects, research institutions and funders need to implement strategies to ensure research continuity.

**Objective:**

To scope the literature on challenges to research continuity and strategies for ensuring research continuity during crises.

**Methods:**

We followed standard scoping review methodology and reported findings in accordance with the Preferred Reporting Items for Systematic Reviews and Meta-Analyses extension for Scoping Reviews (PRISMA-ScR) guidelines. We included quantitative, qualitative, and mixed-methods primary studies on our topic across all research disciplines and types of crises. We searched MEDLINE (OVID) and Scopus databases (January 2000 to January 2026). We abstracted data on study characteristics, challenges, mitigation strategies, and the role of funders. We summarized categorical data descriptively and analyzed qualitative data thematically presenting our findings in narrative and tabular formats.

**Results:**

Out of 22,002 captured citations, we identified 112 eligible studies. Most studies focused on pandemic-related crises (91%), mostly related to clinical research (47%) and social sciences (46%), and were conducted predominantly in high-income countries (80%). Challenges occurred at the project (81%), institutional (27%), and funder (15%) levels. The most common challenges for these levels were respectively project suspensions (58%), lack of institutional support (14%), and poor or no research funding (12%). Key mitigation strategies were flexible research operations (80%), adjustments to institutional policies and governance (25%), and implementing strategic interventions (31%). Funders contributed to research continuity, primarily through financial flexibility (56%) and supporting adaptations (39%).

**Conclusion:**

This scoping review maps the key challenges, strategies, and funder roles in sustaining research during crises. Findings highlight the need for flexible, resilient, and well-coordinated research systems across multiple levels. Future research should evaluate the long-term effectiveness of these strategies.

## Introduction

A crisis is a situation involving “an urgent threat to basic structures or fundamental values” characterized by conditions of uncertainty and the need for a far-reaching response [1,2]. Many researchers operate in crisis settings either because their personal or professional environments are affected by a crisis or because their work focuses on crisis-related topics. Crises can be broadly categorized as natural (e.g., earthquakes and hurricanes) or human-made (e.g., armed conflicts and political unrest) events [3].

Crises can disrupt research by affecting the continuity of research operations and the quality and integrity of findings [4]. The COVID-19 pandemic, for example, exposed global vulnerabilities in research systems: laboratories were closed, fieldwork was halted, and collaboration networks were disrupted due to lockdowns and mobility restrictions [5]. Natural disasters like hurricanes can disrupt research by displacing participants, limiting access to clinical sites, and creating financial constraints [6].

These experiences highlight the urgent need for mechanisms to ensure research continuity during crises [7]. A number of terms have been used to characterize research continuity in this context. ‘Resilience’ is probably the most frequently used term [8]. Abdel Rahman et al. (2021) defined resilience in research as the “ability to adapt and continue research throughout a crisis while maintaining consistency with the overall research design to successfully complete a research project” [7]. ‘Agility’ in research has also been used in this context and is closely related to flexibility, speed, and adaptability in identifying and responding to challenges and uncertainties [9,10].

Organizational resilience reflects an institution’s capacity to identify potential threats, respond to, and learn from crises to sustain essential operations [8]. It is increasingly conceptualized as a dynamic, process-oriented construct encompassing the capacities to anticipate, cope with, and adapt to disruptions [11]. These capacities exist across multiple interconnected levels, enabling adaptive responses in the research environment. To support research continuity, organizations implement strategies such as flexible work policies for researchers, adjustments to research protocols, virtual communication platforms for collaboration, and adaptive leadership practices [4,7,12,13]. Funders can also play a critical role in ensuring research continuity. During the COVID-19 pandemic, grant extensions, budget reallocations, and emergency funding policies helped mitigate disruptions and sustain research productivity [12].

Considering the substantial work that has accumulated on the topic across research disciplines and types of crises, the objective of this study is to scope the literature on challenges to research continuity and strategies for ensuring research operational continuity during crises.

## Methods

This study follows standard scoping review methodology based on an a priori protocol [14] and is reported in accordance with the Preferred Reporting Items for Systematic Reviews and Meta-Analyses extension for Scoping Reviews (PRISMA-ScR) guidelines (S1 File).

### Eligibility criteria

We included studies meeting the following eligibility criteria:

- Study design: articles reporting on quantitative, qualitative, or mixed methods studies. We excluded commentaries, case reports, editorials, opinion pieces, protocols, systematic reviews, literature reviews, and other non-primary research articles.
- Population: either researchers or research organizations from any discipline.
- Topic: (1) challenges encountered by researchers during crises, (2) strategies implemented to promote research operational continuity, and (3) the role of funders in supporting research operational continuity. We considered all types of crises, including but not limited to conflict, pandemics, natural disasters, political instability, or other emergencies that disrupt research activities.
- Setting: no restriction by country or region.
- Language: studies published in English.
- Year of publication: from the year 2000 onwards to capture contemporary data.

### Search strategy

We developed a search strategy in consultation with an information specialist and director of evidence synthesis for MEDLINE and SCOPUS databases. The search was limited to studies published in English from the year 2000 onwards. The initial search was conducted on November 15, 2024, and an updated search was performed on January 15, 2026, prior to final data abstraction to identify any newly published studies. The search strategy combined keywords and controlled vocabulary (e.g., MeSH terms) related to the concepts of “crisis”, “research”, “resilience”, and “organizations” (S1 Appendix). We pilot tested the search strategy using seed articles to refine the final version. In addition, we performed both backward and forward citation tracking on included articles to identify additional eligible studies.

### Article selection

Six reviewers worked in pairs to screen, in duplicate and independently, the titles and abstracts of citations captured by the search for potential eligibility. Screening and full-text review were managed using Covidence software [15]. All reviewers completed calibration exercises before commencing the screening process to ensure consistency and reliability. We retrieved the full texts of all citations judged as potentially eligible by at least one reviewer. Each full text was then screened in duplicate and independently using a standardized screening form. Disagreements between reviewers were resolved through discussion or, when necessary, by consultation with a third reviewer (EAA).

### Data abstraction

We developed and pilot tested a standardized data abstraction form. The form was refined to ensure it captured all relevant information and enabled consistent interpretation among reviewers. Reviewers completed a calibration exercise using a sample of included studies. Pairs of reviewers then independently abstracted data in duplicate and independently on Covidence. Any discrepancies were resolved through discussion or, when necessary, with the involvement of a third reviewer (EAA).

We abstracted data on study general characteristics including study design, type of crisis, research discipline, geographical region, country classification of the crisis-affected setting, and year of publication. Additionally, we abstracted data on challenges encountered by researchers during crises, strategies implemented to promote research operational continuity, and the role of funders in supporting research operational continuity.

### Data analysis

We analyzed qualitative data to identify common themes and used counting to quantify the frequency with which the themes were addressed across included studies. We used ChatGPT (OpenAI, accessed July 2025) to support initial theme generation from the abstracted data. We validated the AI-generated themes through comparison against the abstracted data and refined them to ensure consistency. We categorized challenges according to three levels: the project, the institution, and the funder. We summarized the findings of the included studies in narrative and tabular formats.

**Ethical approval:** The study involves no human subjects and requires no ethical approval.

## Results

Our search retrieved a total of 22,002 studies. After removing duplicates, 18,169 remained for title and abstract screening. A total of 338 studies were retrieved for full-text screening, resulting in 112 studies included in the review. Full-text studies were excluded for the following reasons: wrong topic addressed, wrong population, or wrong study design. Fig 1 demonstrates the inclusion and exclusion of studies at each stage of the screening process.

**Fig 1:**
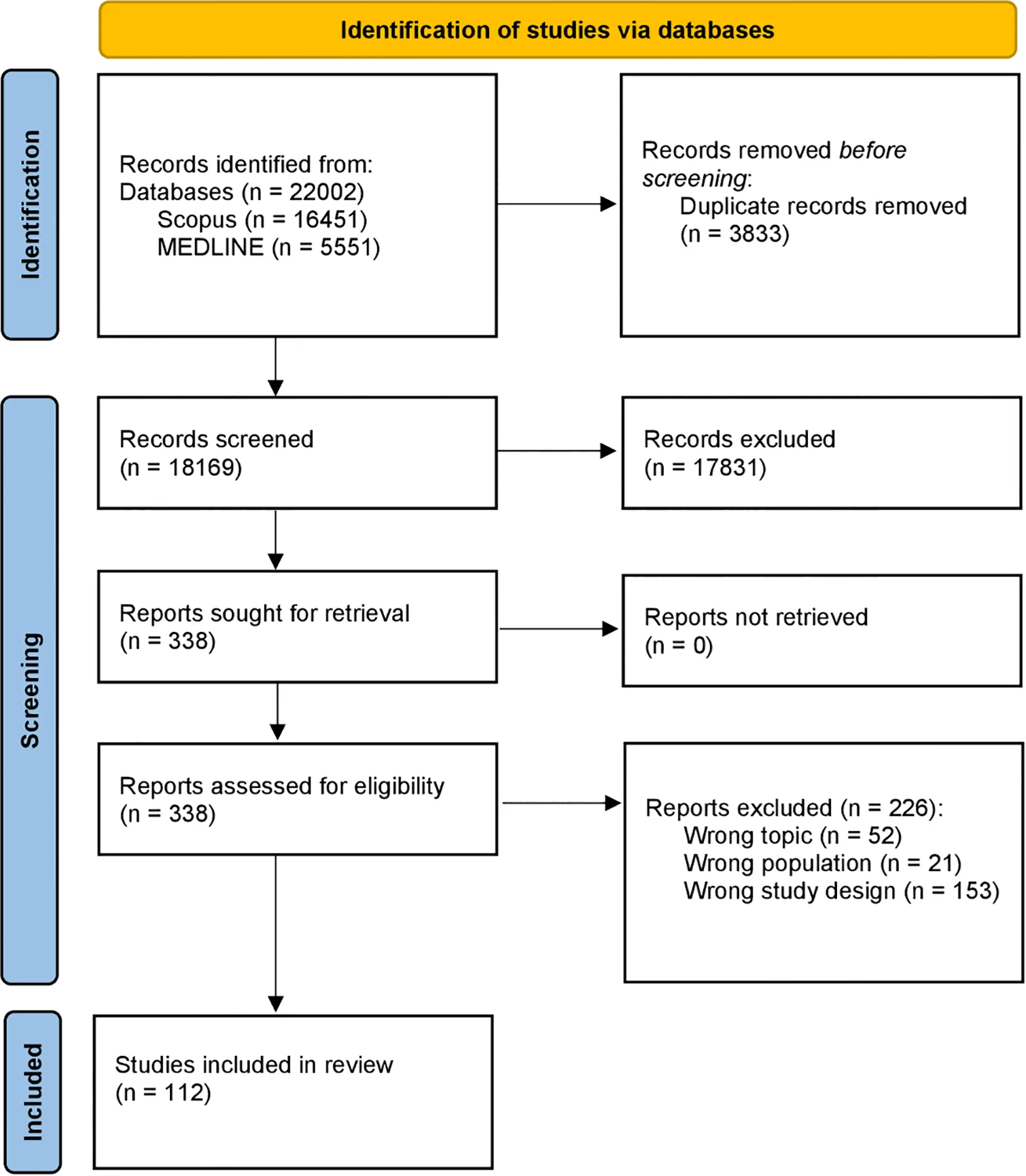
PRISMA-flow Diagram.

### Characteristics of included studies

S2 Appendix outlines the general characteristics of the included studies (n = 112). Nearly half of the primary studies used a mixed-methods design (47%), while the remainder were either quantitative (27%) or qualitative (26%). Most studies focused on crises related to pandemics (91%), and mostly related to clinical field (47%), social sciences (46%), and/or public health (39%). These studies predominantly examined crisis-affected settings in North America (39%) and Europe (33%) and were mostly conducted in countries classified as high-income (80%). The median year of publication was 2022 (IQR: 2021–2023).

### Challenges in ensuring research continuity

S3 Appendix provides the detailed results for the challenges, mitigation strategies, and the role of funders in ensuring research continuity. Fig 2 provides a summary of these findings.

**Fig 2:**
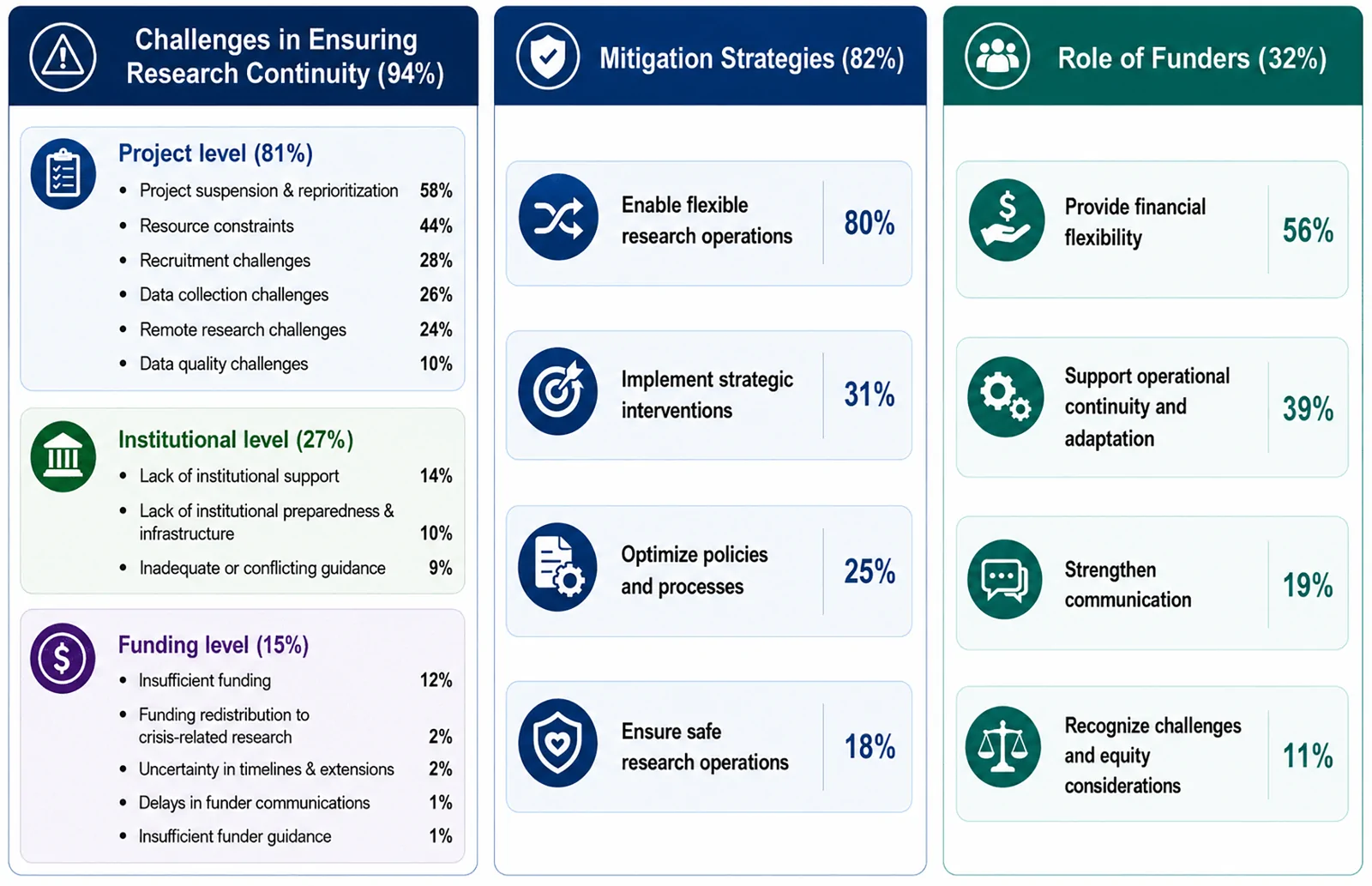
Summary of challenges, mitigation strategies and the role of funders in ensuring research continuity.

One-hundred-and-five studies (94%) reported on challenges in ensuring research continuity during crises. These one-hundred-and-five studies addressed challenges at either the project (81%), institutional (27%), or the funder levels (15%).

At the project level, the most frequently reported challenge was project suspension and reprioritization (58%), primarily due to the cancellation or slowing down of research activities (29%). Resource constraints was also a common challenge (44%), largely related to limited access to infrastructure and equipment (29%). Challenges in participant recruitment (28%) were mainly due to reduced recruitment linked to participant fear, illness, or death (20%), while data collection challenges (26%) were predominantly associated with delays or cancellations of data collection activities (13%). Remote research challenges (24%) were most often related to technological difficulties, including limited familiarity with digital tools and unstable internet connections (15%). Additional challenges included data quality issues (10%), primarily reflecting lower data quality (7%).

At the institutional level, the most frequently reported challenges was the lack of institutional support (14%), mainly due to poor leadership engagement (11%). This was followed by a lack of preparedness and infrastructure (10%), often linked to infrastructural breakdowns such as internet or electricity disruptions (5%). Inadequate or conflicting guidance (9%) further hindered research continuity, particularly due to vague or insufficient institutional policies (5%).

At the funder level, challenges were primarily related to poor or no research funding (12%), alongside issues such as redistribution of funding toward crisis-related research (2%) and uncertainty in funding timelines (2%). Delays in funder communication and insufficient guidance were less frequently reported (1%).

### Mitigation strategies to ensure research continuity

Ninety-one studies (82%) reported strategies to mitigate disruptions and support research continuity during crises (S3 Appendix). These included ensuring safe research operations, introducing flexibility into research operations, optimizing policies and processes, and implementing strategic interventions.

Measures to ensure safe research operations (18%) included implementing hygiene standards and social distancing measures (9%), alongside contingency planning to support continuity of essential research activities (9%).

Introducing flexibility into research operations was the most frequently reported strategy (80%). It included transitioning research processes to virtual modalities (57%); providing training to support adaptation to remote methods (25%) and investing in digital infrastructure (11%).

Optimizing policies and processes (25%) included adjusting work conditions related policies (14%), issuing clear and updated guidance (8%) and adapting ethics review processes (3%).

Finally, strategic interventions (31%) included developing new partnerships (10%), promoting interdisciplinary learning and reflection (9%), strengthening existing collaborations through improved coordination and shared resources (7%), and building trust and collaboration between leadership and staff (4%).

### Role of funders in ensuring research continuity

Thirty-six studies (32%) reported on the role of funders in supporting research continuity during crises (S3 Appendix). Funders were commonly reported to provide financial flexibility (56%) through funding extensions (25%), budget reallocations (22%) and increases in overall funding to address emerging needs (22%). Also, they supported operational continuity (39%), by permitting procedural and methodological adjustments to research protocols (17%) and providing administrative and logistical support (8%). Less frequently reported approaches included strengthening communication (19%) and recognizing challenges and equity considerations (11%).

## Discussion

### Summary of findings

This scoping review identified one-hundred-and-twelve studies reporting on challenges (94%), mitigation strategies (82%), and the role of funders (32%) in ensuring research continuity during crises. Challenges were reported at the project, institutional, and funder levels. At the project level, the most frequently reported challenges included disruptions to research activities, resource constraints, and difficulties in participant recruitment and data collection. At the institutional level, challenges were most commonly related to limited support, lack of preparedness, and inconsistent or unclear guidance. At the funder level, insufficient or unstable funding and delays in communication were the main challenges.

To address these challenges, the most commonly reported mitigation strategies were the adoption of flexible research operations, implementation of strategic interventions, and adjustments to institutional policies and processes. The role of funders primarily involved providing financial flexibility and supporting operational continuity and adaptations.

### Strengths and limitations

Our review identified a variety of up-to-date studies examining challenges to, and strategies for, ensuring research continuity during crises. We developed an a priori protocol and followed standard scoping review methodology, placing no restrictions on the types of crises addressed or research disciplines.

In terms of limitations, we restricted our search strategy to studies published in English. Also, the included studies might not be optimally representative as many focused on pandemic-related crises and were conducted in high-income countries. This potentially limits the generalizability of findings to other types of crises, particularly economic, political, and environmental crises, and to low- and middle-income settings where challenges and mitigation strategies may differ.

### Comparison to similar research

We identified two narrative reviews on challenges in clinical research during the COVID-19 pandemic [16,17]. Nomali et al, and similarly to our study, identified disruptions to participant recruitment, data quality, and ethical concerns [16]. Kofi et al.’s highlighted multidimensional challenges including limited access to laboratories, supply chain disruptions, and ethical challenges during pandemics [17]. Both studies highlighted strategies advocating for remote methods, digital infrastructure, and flexible ethical frameworks. We also identified a scoping review by Sigfrid et al. on clinical research responses to epidemics and pandemics [18]. It highlighted fragmented coordination, delays in funding and approvals, ethical complexities, and infrastructural limitations, and recommended stronger collaborations, pre-approved protocols, and community engagement. [18]. While these studies primarily focus on clinical research in pandemics, our scoping review extends this perspective across multiple types of crises, a broader range of research domains, and different levels.

### Implications for practice

Our findings highlight the need to build resilient and well-coordinated research systems capable of maintaining operational continuity during crises. Institutions should prioritize flexible research operations, including the rapid transition to remote research methods. This requires investment in digital infrastructure, such as reliable internet access, secure platforms, digital tools, and access to software, as well as training to support researchers in using these tools effectively.

Flexible governance and ethical oversight are also essential. Institutions should optimize policies and processes to better support researchers during crises. This includes the development of responsive governance structures that enable timely decision-making, minimize delays, and support adaptive ethics review procedures. Strengthening collaboration among research teams, institutions, funders, and communities further enhances system responsiveness and ensures that research remains relevant and effective during crises. Funders can also support research continuity through financial flexibility, including funding extensions and adaptive funding arrangements, as well as logistical support.

## Conclusion

Ensuring research continuity during crises requires a multi-level, proactive approach that spans researchers, institutions, and funding agencies. With the predominance of literature on COVID-19, future research should evaluate the effectiveness of the identified strategies across different types of crises, including economic, political, and environmental crises, and across diverse settings, particularly in low- and middle-income countries, to guide evidence-based preparedness planning. Future efforts could focus on developing practical guidance for research continuity, translating the findings into actionable recommendations for researchers, institutions, and funders during crises.

## Supporting information

S1 File PRISMA-ScR checklist. (DOCX)

S1 Appendix Search strategy databases. (DOCX)

S2 Appendix General characteristics of included studies. (DOCX)

S3 Appendix Challenges, mitigation strategies, and the role of funders in ensuring research continuity. (DOCX)
