# Supplementary material for: Ensuring research continuity during crises: A scoping review
Source: PLoS One. 2026 Nov 2;21(11):e0353082. doi: 10.1371/journal.pone.0353082 (PMC13726960; doi:10.1371/journal.pone.0353082)
Supplement: Supplementary file 2 [file pone.0353082.s002.docx]

**S1 Appendix: Search Strategy for Databases**

**Medline**

**1**  Coping Skills/ or Survivorship/ or Survivors/ or Social Vulnerability/ or Social Support/
**2**  (resilien* or coping* or cope?).ti,ab.
**3**  Research/ or Animal Experimentation/ or Behavioral Research/ or Biobehavioral Sciences/ or Biomedical Research/ or Health Services Research/ or Human Experimentation/ or Interdisciplinary Research/ or Qualitative Research/ or Research Design/ or Research Personnel/ or Community-Based Participatory Research/ or Public Health Systems Research/ or Dental Research/ or Empirical Research/ or Genetic Research/ or Nursing Education Research/ or Nursing Research/ or Peer Review Research/ or Clinical Nursing Research/ or Ethics, Research/ or Pharmaceutical Research/ or Pharmacy Research/ or Rehabilitation Research/ or Translational Research, Biomedical/ or Epidemiologic Research Design/ or Embryo Research/ or Fetal Research/ or Nursing Evaluation Research/ or Nursing Methodology Research/ or Social Validity, Research/ or Space Research/ or Stem Cell Research/ or Operations Research/ or Nursing Administration Research/
**4**  (research* or experiment*).ti,ab.
**5**  ethnic cleansing/ or terrorism/ or bioterrorism/ or chemical terrorism/ or mass casualty incidents/ or "warfare and armed conflicts"/ or armed conflicts/ or warfare/ or biological warfare/ or chemical warfare/ or nuclear warfare/ or psychological warfare/ or war crimes/ or genocide/ or holocaust/ or war exposure/ or war-related injuries/ or Emergencies/ or Economic Recession/ or disasters/ or natural disasters/ or avalanches/ or cyclonic storms/ or droughts/ or earthquakes/ or floods/ or landslides/ or tornadoes/ or wildfires/ or explosions/ or fires/ or disease outbreaks/ or disease hotspot/ or epidemics/ or pandemics/ or endemic diseases/ or nuclear weapon/ or bombs/ or CIVIL DISORDERS/ or economic stability/
**6**  Blast Injuries/ or gun violence/ or mass shooting events/ or tidal waves/ or afghan campaign 2001-/ or gulf war/ or iraq war, 2003-2011/ or korean war/ or september 11 terrorist attacks/ or world war i/ or world war ii/ or Riots/ or disaster planning/ or pandemic preparedness/ or american civil war/ or american revolution/ or crimean war/ or french revolution/ or russian-japanese war/ or sino-japanese war/ or spanish-american war, 1898/ or vietnam conflict/
**7**  (warfare or war or wars or genocid* or ethnocid* or te?rori* or biote?rori* or biocrim* or biowar* or bio-war* or bio-te?rori* or bio-crim* or holocaust* or war-related or crime-war* or (conflict* adj2 (setting* or area* or region* or countr* or city or cities or situation* or armed or ethnic)) or (mass adj1 (casualt* or disaster*)) or ((financ* or econom* or money or politic*) adj2 (crisis or crises or rece?sion* or collaps* or downturn or instabilit*)) or disaster* or catastroph* or earthquake* or earth-quake* or avalan?h* or cyclon* or hu?ricane* or typhoon* or landslide* or (land adj slide) or tornado* or tsunami* or explos* or blast or blasts or bomb* or pandemic* or epidemic* or fire or fires or wildfire* or wild-fire* or ((disease? or infect*) adj3 (outbreak* or cluster* or endemic* or hotspot*)) or cris?s or agility).ti,ab.
**8**  (((nuclear adj weapon*) or (ethnic or race or races or racial)) adj cleansing*).ti,ab.
**9**  ((burden or epidemiolg* or transmission*) adj3 hotspot).ti,ab.
**10**  (civil adj (unrest or disorder* or disturb*)).ti,ab.
**11**  ((mass or communit* or societ* or group?) adj (casualt* or incident* or shoot* or violen* or trauma* or murder* or kill or killed or killing)).ti,ab.
**12**  ((tide* adj wave?) or riot* or (gas* adj poisoning*) or (gun adj violence)).ti,ab.
**13**  5 or 6 or 7 or 8 or 9 or 10 or 11 or 12
**14**  3 or 4
**15**  Organizations/ or "Academies and Institutes"/ or Government Agencies/ or Organizations, Nonprofit/ or Universities/ or Hospitals/
**16**  (organi?ation or organi?ations or center or centers or centre or centres or universit* or hospital or hospitals or institution or institutions or academy or academies or institute or instituites).ti,ab.
**17**  15 or 16
**18**  ((manag* or flexibile* or flexibilit* or ac?limitiz* or control* or surviv* or support* or continu* or adapt* or re-adapt* or readapt* or adjust*) adj5 research*).ti,ab.
**19**  1 or 2 or 18
**20**  13 and 14 and 17 and 19
**21**  limit 20 to (english language and yr="2000 - 2025")

**Scopus**

( TITLE-ABS ( ( research* OR experiment* ) ) ) AND ( TITLE-ABS ( ( organisation OR organisations OR organization OR organizations OR center OR centers OR centre OR centres OR universit* OR hospital OR hospitals OR institution OR institutions OR academy OR academies OR institute OR instituites ) ) ) AND ( ( TITLE-ABS ( ( resilien* OR coping* OR cope OR copes ) ) ) OR ( TITLE-ABS ( ( ( manag* OR flexibile* OR flexibilit* OR aclimitiz* OR acclimitiz* OR control* OR surviv* OR support* OR continu* OR adapt* OR re-adapt* OR readapt* OR adjust* ) W/4 research* ) ) ) ) AND ( ( TITLE-ABS ( ( ( tide* PRE/0 ( wave OR waves ) ) OR riot* OR ( gas* PRE/0 poisoning* ) OR ( gun PRE/0 violence ) ) ) ) OR ( TITLE-ABS ( ( warfare OR war OR wars OR genocid* OR ethnocid* OR terrori* OR terori* OR bioterori* OR bioterrori* OR biocrim* OR biowar* OR bio-war* OR bio-terori* OR bio-terrori* OR bio-crim* OR holocaust* OR war-related OR crime-war* OR ( conflict* W/1 ( setting* OR area* OR region* OR countr* OR city OR cities OR situation* OR armed OR ethnic ) ) OR ( mass W/0 ( casualt* OR disaster* ) ) OR ( ( financ* OR econom* OR money OR politic* ) W/1 ( crisis OR crises OR recession* OR recesion* OR collaps* OR downturn OR instabilit* ) ) OR disaster* OR catastroph* OR earthquake* OR earth-quake* OR avalanch* OR avalansh* OR cyclon* OR huricane* OR hurricane* OR typhoon* OR landslide* OR ( land PRE/0 slide ) OR tornado* OR tsunami* OR explos* OR blast OR blasts OR bomb* OR pandemic* OR epidemic* OR fire OR fires OR wildfire* OR wild-fire* OR ( ( disease OR diseases OR infect* ) W/2 ( outbreak* OR cluster* OR endemic* OR hotspot* ) ) OR crisis OR crises OR adversity ) ) ) OR ( TITLE-ABS ( ( ( ( nuclear PRE/0 weapon* ) OR ( ethnic OR race OR races OR racial ) ) PRE/0 cleansing* ) ) ) OR ( TITLE-ABS ( ( ( burden OR epidemiolg* OR transmission* ) W/2 hotspot ) ) ) OR ( TITLE-ABS ( ( civil PRE/0 ( unrest OR disorder* OR disturb* ) ) ) ) OR ( TITLE-ABS ( ( ( mass OR communit* OR societ* OR group OR groups ) W/0 ( casualt* OR incident* OR shoot* OR violen* OR trauma* OR murder* OR kill OR killed OR killing ) ) ) ) ) AND PUBYEAR > 1999 AND PUBYEAR < 2026 AND PUBYEAR > 1999 AND PUBYEAR < 2026 AND ( LIMIT-TO ( LANGUAGE , "English" ) )
