# Supplementary material for: Ensuring research continuity during crises: A scoping review
Source: PLoS One. 2026 Nov 2;21(11):e0353082. doi: 10.1371/journal.pone.0353082 (PMC13726960; doi:10.1371/journal.pone.0353082)
Supplement: Supplementary file 3 [file pone.0353082.s003.docx]

**S2 Appendix: General characteristics of included studies (n=112)**

|  | n (%)* |
| --- | --- |
| Study design |  |
| *Mixed-methods* | 53 (47) |
| *Quantitative* | 30 (27) |
| *Qualitative* | 29 (26) |
| Type of crisis |  |
| *Pandemic* | 102 (91) |
| *Conflict* | 7 (6) |
| *Natural disaster* | 3 (3) |
| *Political* | 3 (3) |
| *Financial* | 1 (1) |
| Research Discipline |  |
| *Clinical* | 53 (47) |
| *Social sciences* | 52 (46) |
| *Public health* | 44 (39) |
| *STEM* | 22 (20) |
| *Humanities* | 11 (10) |
| *Other*** | 13 (12) |
| Geographical region of the crisis-affected setting |  |
| *North America* | 44 (39) |
| *Europe* | 37 (33) |
| *East Asia & Pacific* | 18 (16) |
| *Africa* | 15 (13) |
| *South Asia* | 10 (9) |
| *Middle East* | 8 (7) |
| *Latin America* | 6 (5) |
| Country classification of the crisis-affected setting^1^ |  |
| *High-income* | 90 (80) |
| *Upper middle-income* | 13 (12) |
| *Lower middle-income* | 17 (15) |
| *Lower income* | 3 (3) |
| Year of publication (median, IQR) | 2022 (2021 – 2023) |
| * Percentages may not add up to 100 due to rounding and non-mutually exclusive categories  ** Other: Environmental sciences (5%), Translational sciences (2%), General (2%), Interdisciplinary (2%), Agricultural sciences (1%)  ^1^According to the World Bank classification (<https://datahelpdesk.worldbank.org/knowledgebase/articles/906519-world-bank-country-and-lending-groups>) | |
