# Supplementary material for: Ensuring research continuity during crises: A scoping review
Source: PLoS One. 2026 Nov 2;21(11):e0353082. doi: 10.1371/journal.pone.0353082 (PMC13726960; doi:10.1371/journal.pone.0353082)
Supplement: Supplementary file 4 [file pone.0353082.s004.docx]

**S3 Appendix: Challenges, mitigation strategies, and the role of funders in ensuring research continuity (n=112)**

1. **Challenges in ensuring research continuity (n=105, 94%)**

- Project level
- Institutional level
- Funding level

**Project level (n=85, 81%)**

Project suspension and reprioritization (n = 61, 58%)

- Cancellation or slowing down of research activities (n = 30, 29%)
- Reduction in project productivity (n = 14, 13%)
- Suspension of interdisciplinary/collaborative work (n = 9, 9%)
- Pausing or ending non–crisis related studies to prioritize crisis related research (n = 7, 7%)
- Uncertainty about project duration and rapid changes (n = 5, 5%)

Resource constraints (n = 46, 44%)

- Limited access to infrastructure and equipment (e.g., laboratories, offices, libraries, archives, and software needed for research) (n = 30, 29%)
- Personnel shortages due to clinical workload, staff redeployment, illness, fear of transmission, and caregiving responsibilities (n = 15, 14%)
- High costs for digital data collection devices and training (n = 4, 4%)

Participant recruitment challenges (n = 29, 28%)

- Reduction in recruitment due to protective measures or participant fear, illness, or death (n = 21, 20%)
- Ethical concerns related to participant vulnerability and therapeutic misconception (n =4, 4%)
- Difficulty reaching underrepresented, digitally underserved, or rural populations (n = 4, 4%)

Data collection challenges (n = 27, 26%)

- Delays or cancellations of data collection activities (n =14, 13%)
- Inability to conduct in-person fieldwork (n = 11, 10%)
- Difficulty following up on participants (n = 4, 4%)

Remote research challenges (n = 25, 24%)

- Technological difficulties (e.g., staff/participant lack of familiarity with digital tools, unstable internet connection at home) (n = 16, 15%)
- Communication difficulties (e.g., interpreting non-verbal cues, discussing sensitive topics remotely) (n = 5, 5%)
- Procedural difficulties (e.g., trust-building, informed consent, privacy) (n = 4, 4%)

Data quality challenges (n = 11, 10%)

- Lower data quality (n = 7, 7%)
- Reduction in sample sizes (n =4, 4%)

**Institutional level (n=28, 27%)**

Lack of institutional support (n = 15, 14%)

- Poor institutional and leadership support (e.g., denying access to relevant data, feelings of disconnection, limited appreciation of researcher demands and workload) (n = 12, 11%)
- Power imbalances and top-down decision-making approaches (n = 3, 3%)
- Fear of voicing concerns, opinions, or requests to leadership (n = 1, 1%)

Lack of institutional preparedness and infrastructure (n = 10, 10%)

- Infrastructural breakdowns (e.g., pressure on local internet and electricity services) (n = 5, 5%)
- Lack of preparedness for crises (e.g., no contingency plans, no electronic submission processes) (n = 5, 5%)

Inadequate or conflicting guidance (n = 9, 9%)

- Vague or insufficient institutional guidance/policies (n = 5, 5%)
- Conflicts between institutional and local guidance (n = 4, 4%)
- Safety protocols not tailored to specific research needs or contexts (n = 1, 1%)
- Slow communication from upper administration (n = 1, 1%)

**Funding level (n=16, 15%)**

- Poor or no research funding (n = 13, 12%)
- Redistribution of funding toward crisis-related research (n = 2, 2%)
- Uncertainty in funding timelines and extensions (n = 2, 2%)
- Delays in funder communications (n = 1, 1%)
- Insufficient guidance from funding agencies (e.g. permission to pay staff from grant funding) (n = 1, 1%)

2. **Mitigation Strategies (n=92, 82%)**

- Ensure safe research operations
- Enable flexible research operations
- Optimize policies and processes
- Implement strategic interventions

**Ensure safe research operations (n = 16, 18%)**

- Implement hygiene standards, social distancing, and facility management systems to minimize exposure risks (n = 8, 9%)
- Implement contingency planning to triage research activities and provide alternative access/support methods (e.g., by conducting risk assessments and audits) (n = 8, 9%)

**Enable flexible research operations (n = 73, 80%)**

- Transition research processes (e.g., recruitment, data collection, monitoring) to virtual modalities (e.g., crowd-sourcing, telephone calls, video conferencing, telemedicine, e-consent) (n = 52, 57%)
- Provide training modules (e.g., short courses or online learning) to support adaptation to remote research methods, promote equity, and strengthen collaboration (n = 23, 25%)
- Invest in digital infrastructure (e.g., internet, digital libraries, laptops, and software licenses) (n = 10, 11%)
- Allow flexibility in remote data collection protocols (n = 7, 8%)
- Permit remote work options and asynchronous participation in meetings (n = 6, 7%)
- Use secondary data sources (e.g., from other centers, previously collected data) (n = 5, 5%)

**Optimize policies and processes (n = 23, 25%)**

- Adjust policies to support researchers (e.g., caregiving responsibilities, protected research time) (n = 13, 14%)
- Issue clear, timely, and updated guidance aligned with local conditions and research needs (n = 7, 8%)
- Adjust ethics review timelines and procedures (e.g., e-consent, fast-track approvals, virtual submissions and meetings) (n = 3, 3%)

**Implement strategic interventions (n = 28, 31%)**

- Foster new partnerships in public health, digital health, and pandemic preparedness including engagement with community gatekeepers (n = 9, 10%)
- Foster collective and inter-disciplinary learning and reflection through facilitated online discussions and forums (n = 8, 9%)
- Strengthen existing collaborations through shared funding calls or improved coordination processes (e.g. with ethics reviewers to optimize protocol review timelines, or with funders to enable timely support) (n = 6, 7%)
- Build trust and sympathy between management and staff across disciplines for rapid decision-making and innovation (n=4, 4%)
- Reallocate internal funds to maintain ongoing projects and staff salaries (n = 4, 4%)
- Designate individuals to communicate cross-departmental feedback to higher authorities for timely solutions (n = 2, 2%)
- Create consortium to share data, tools, and resources more efficiently (n = 2, 2%)
- Establish feasibility committees to review, triage, and prioritize crisis-related research initiatives aligned with national research mandates (n = 1, 1%)

3. **Role of funders in ensuring research continuity (n=36, 32%)**

- Provide financial flexibility
- Support operational continuity and adaptation
- Recognize challenges and equity considerations
- Strengthen communication

**Provide financial flexibility (n = 20, 56%)**

- Provide funding extensions to protect ongoing projects and research staff (n = 9, 25%)
- Allow budget reallocation flexibility (e.g., shifting funds across budget lines or consortium partners to support remote research) (n = 8, 22%)
- Increase overall funding during crisis periods to meet emerging needs (n = 8, 22%)

**Support operational continuity and adaptation (n = 14, 39%)**

- Allow procedural and methodological adjustments to protocols (e.g., research design, questions, timelines, and community involvement) (n = 6, 17%)
- Provide administrative and logistical support (e.g., donation of research supplies, flexibility in salaries, support access to materials, datasets, or software remotely) (n = 3, 8%)
- Support the postponement or reprioritization of studies based on feasibility and risk (n = 3, 8%)
- Support research infrastructure and costs (e.g., research centers, units, and open access publication fees) (n = 2, 6%)

**Recognize challenges and equity considerations (n = 4, 11%)**

- Recognize and address researcher challenges (e.g., hardship funds to cover long-term costs of cancelled fieldwork) (n = 3, 8%)
- Promote visibility of opportunities to support researchers with specific needs (e.g., caregivers, students with second jobs) (n = 1, 3%)

**Strengthen communication (n = 7, 19%)**

- Provide faster and clearer communication on acceptable adaptations (e.g., through funder-implementer collaboration) (n = 7, 19%)
